# Evaluating the Safety and Usability of an Over-the-Counter Medical Device for Adults With Mild to Moderate Hearing Loss: Formative and Summative Usability Testing

**DOI:** 10.2196/65142

**Published:** 2025-01-20

**Authors:** Megan Elizabeth Salwei, Shilo Anders, Carrie Reale, Jason M Slagle, Todd Ricketts, Matthew B Weinger

**Affiliations:** 1 Center for Research and Innovation in Systems Safety, Department of Anesthesiology, Vanderbilt University Medical Center, 2525 West End Avenue, Suite 800, Nashville, TN, 37203, United States, 16153431528; 2 Department of Hearing and Speech Sciences, Vanderbilt University Medical Center, Nashville, TN, United States

**Keywords:** usability, human factors, patient safety, over-the-counter hearing aids, direct-to-consumer hearing aids, medical device, hearing loss, adult, hearing impairment, hearing aid use, hearing care, formative usability test, safety, mobile phone

## Abstract

**Background:**

Only 15% of the nearly 30 million Americans with hearing loss use hearing aids, partly due to high cost, stigma, and limited access to professional hearing care. Hearing impairment in adults can lead to social isolation and depression and is associated with an increased risk of falls. Given the persistent barriers to hearing aid use, the Food and Drug Administration issued a final rule to allow over-the-counter hearing aids to be sold directly to adult consumers with perceived mild to moderate hearing loss at pharmacies, stores, and online retailers without seeing a physician or licensed hearing health care professional.

**Objective:**

We evaluated the safety and usability of an over-the-counter hearing aid prior to Food and Drug Administration approval and market release.

**Methods:**

We first conducted a formative usability test of the device and associated app with 5 intended users to identify outstanding safety and usability issues (testing round 1). Following design modifications, we performed a summative usability test with 15 intended users of the device (testing round 2). We concurrently conducted a test with 21 nonintended users (ie, users with contraindications to use) to ascertain if consumers could determine when they should not use the device, based on the packaging, instructions, and labeling (testing round 3). Participants were asked to complete 2‐5 tasks, as if they were using the hearing aid in real life. After each task, participants rated the task difficulty. At the end of each session, participants completed a 10-question knowledge assessment and the System Usability Scale and then participated in debriefing interviews to gather qualitative feedback. All sessions were video recorded and analyzed to identify use errors and design improvement opportunities.

**Results:**

Usability issues were identified in all 3 usability testing rounds. There were minimal safety-related issues with the device. Round 1 testing led to several design modifications which then increased task success in round 2 testing. Participants had the most difficulty with the task of pairing the hearing aids to the cell phone. Participants also had difficulty distinguishing the right and left earbuds. Nonintended users did not always understand device contraindications (eg, tinnitus and severe hearing loss). Overall, test findings informed 9 actionable design modifications (eg, clarifying pairing steps and increasing font size) that improved device usability and safety.

**Conclusions:**

This study evaluated the usability and safety of an over-the-counter hearing aid for adults with mild to moderate hearing loss. Human factors engineering methods identified opportunities to improve the safety and usability of this direct-to-consumer medical device for individuals with perceived mild-moderate hearing loss.

## Introduction

Approximately 1 in 4 people older than 12 years of age in the United States have hearing loss in 1 or both ears [1], and 30 million American adults could benefit from hearing aid use [2]. Hearing loss is associated with an increased risk for falls [3] and may lead to cognitive decline [4] and dementia [5]. Yet, only 1 in 3 older adults [6] and 15% of all adults with hearing loss use hearing aids [7]. Some barriers preventing adoption include lack of access, stigma, and financial challenges with traditional hearing aids costing between US $1000 and US $6000 per ear [8]. Aiming to mitigate these barriers, the Food and Drug Administration (FDA) issued a final rule in 2022 [9] permitting the sale of hearing aid devices without a prescription or medical exam. Yet, there are few studies of the usability and safety design challenges of over-the-counter medical devices intended to be sold widely in consumer markets.

Over-the-counter or direct-to-consumer medical devices do not require a prescription and can be sold at retail outlets including pharmacies, big-box stores, and online. Examples include self-monitoring blood glucose test systems and at-home pregnancy tests. Typically used outside of health care settings, these devices must have (1) low potential for misuse; (2) benefits that outweigh their safety risks; and (3) sufficient labeling so that lay-users can self-diagnose their condition, self-determine that the device is appropriate for their condition, and understand how to use the device without clinician assistance or instruction [10]. To minimize use errors and their associated risks, the FDA requires premarket review of the safety and effectiveness for most direct-to-consumer medical devices. This review process includes rigorous human factors usability testing with representative users to validate that the warnings, cautions, and contraindications are transparent, assess if users can complete critical tasks of the device, and identify areas of foreseeable misuse (including use of the device by unintended users) [11].

Numerous studies have evaluated the safety and usability of clinician-facing medical devices (eg, smart intravenous infusion pumps) [12-15], as well as patient-facing medical devices (eg, glucometers, ventilators, and epinephrine injectors) [16]. Typically, clinicians still mediate the use of these patient-facing medical devices either by providing a prescription to use the device or instructions on how to use the device appropriately [17]. There remains limited research on the safety and usability of direct-to-consumer medical devices, especially those used by underserved populations (eg, people with hearing impairment) [18-20].

Compared to clinician-facing medical devices, patient-facing devices present a challenge to designers as the potential environment of use and characteristics of end users is much more diverse [16]. The usability of direct-to-consumer devices is especially important given the lack of clinical supervision to support appropriate use. For instance, over-the-counter hearing aids will be on the shelf at consumer electronics stores next to other, nonmedical devices (eg, wireless headphones) and consumers may come across the device without previously identifying a need for a hearing aid. While the risk is minimal, use of hearing aids by those with normal hearing could result in noise-induced hearing loss. In this context, the packaging of the device and communication of its intended use (and contraindications to use such as an ear infection, tinnitus, or severe hearing loss) is especially important. In this study, we evaluated the safety and usability of an over-the-counter hearing aid among adults in the United States.

## Methods

### Ethical Considerations

We conducted usability testing of an over-the-counter hearing aid to evaluate the safety and usability of the device prior to market release. This study was approved by the Vanderbilt University Medical Center’s institutional review board (201894). At the start of each session, we reviewed the consent document with participants and answered any questions they had. All participants provided written consent to be in the study. Data have been deidentified.

### Hearing Aid

**Figure 1.**
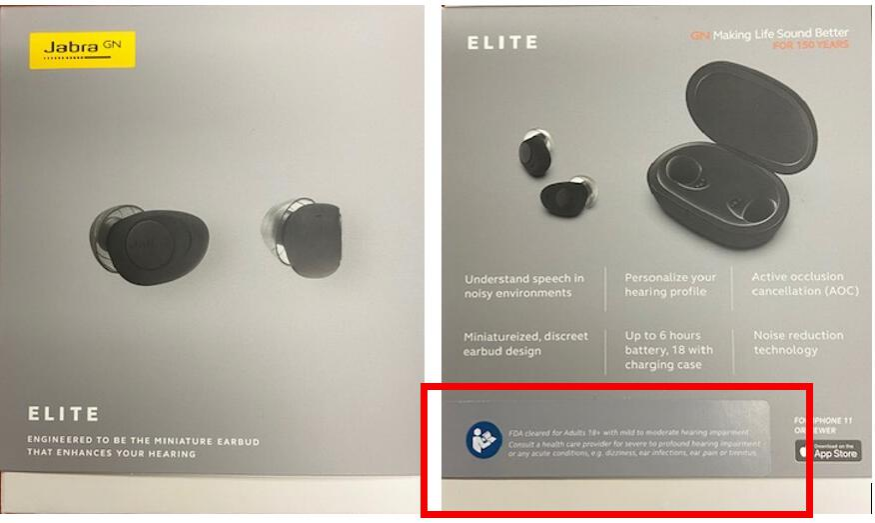
Front and back of the device box.

We evaluated the Jabra Enhance Plus (formerly called the “Jabra Elite” during the study), an over-the-counter hearing aid that is intended to enhance hearing but can also be used for phone calls and listening to music. Users can purchase these hearing aids at a suggested retail price of US $799 per pair. The hearing aids are physically similar to wireless earbuds with adjustable ear gels. Users can pair them to a smartphone and personalize their own hearing aid profile using a free smartphone app (ie, Jabra Enhance app). The miniaturized in-the-ear style earbuds (see Figure 1) come in a small charging case and can be used up to 12 hours on a single charge.

### Participants and Setting

We conducted usability testing with production equivalent prototype devices (ie, packaging, hearing aids, charging case, and user manual) including an iPhone with the Jabra Enhance app installed. We only used iPhones in the usability testing since the Jabra Enhance app was only available for iPhones at the time of testing. We recruited two participant populations for the study, (1) intended users of the device and (2) nonintended users (Textbox 1). We identified potential participants meeting our study criteria through medical record chart review, university newsletters, and word of mouth. Eligible participants conducted a prescreening questionnaire, via phone call or email, to verify they met the inclusion criteria for the study before scheduling. The testing occurred from December 2020 to August 2021.

Textbox 1. **Eligibility criteria for intended and nonintended users.**

Intended users (adults aged 18+) with:

- Mild to moderate hearing impairment

Nonintended users (adults aged 18+) with:

- Normal hearing,
- Severe to profound hearing impairment, and/or
- Acute conditions including tinnitus, dizziness, ear infections, and ear pain

### Intended Users

To start, we conducted usability testing with 5 intended users of the device. Based on the safety and usability issues identified in the testing round (round 1), we made recommendations for modifications to the device packaging and app screens. We then conducted round 2 of usability testing of the revised product with 15 intended users. In each usability testing session, participants were asked to complete 5 tasks (see Textbox 2) within 90 minutes. Participants received a US $50 gift card at the end of the session.

Textbox 2. **Task descriptions.**

**Tasks for intended users (rounds 1 and 2)**

- Read outer label and indicate if use is appropriate
- Set up the hearing aids and app including

-   Find and open hearing aid app
-   Register product and accept terms and conditions
-   Pair the hearing aids
-   Finalize the set up of the hearing aids
-   Fit the hearing aids into ear

- Recharge the hearing aids
- Clean the hearing aids

**Tasks for nonintended users (round 3)**

- Read outer label and indicate if use is appropriate
- Read the inner box contents (note that the inner box contents were available to the participants, but they did not receive explicit instructions on how to read them) and indicate if use is appropriate

### Nonintended Users

We conducted a third round of usability testing with 21 nonintended users of the devices to evaluate if consumers could ascertain that they should not use the device based on the external and internal labeling, user manual, and app screens. We recruited participants who were contraindicated to use the device including adults with normal hearing, adults with severe to profound hearing loss, and adults with tinnitus or severe dizziness. Round 3 participants were scheduled for a 30-minute test session, in which they were asked to complete 2 tasks (see Textbox 2). Participants received a US $20 gift card at the end of the session.

### Data Collection

We conducted functional simulated use studies with end users completing real-world tasks. One human factors engineer facilitated the sessions. In line with real-world use of the device, participants received no training prior to testing. We read task instructions out loud while displaying the same instructions on a computer monitor with closed captioning. Participants were directed to complete 5 (rounds 1 and 2) or 2 tasks (round 3, see Textbox 2). After each task, participants rated how easy or difficult the task was to complete on a scale from 1 (very difficult) to 5 (very easy). After all tasks were completed, we asked intended and nonintended users 10 questions to assess their knowledge of the device and its contraindications for use (see Multimedia Appendix 1). Participants then completed the System Usability Scale (SUS), a 10-item validated survey for assessing the usability of interactive systems [21] (see Multimedia Appendix 2). To conclude the session, we conducted debrief interviews to gather additional qualitative feedback about the safety and usability of the device. The interview guide can be found in Multimedia Appendix 3. All sessions were video recorded and uploaded to Morae (TechSmith), a software suite for usability testing that supports video annotation (eg, of use errors) and logging of tasks.

### Data Analysis

One researcher coded the videos in Morae, annotating areas of difficulty, confusion, safety-related issues, and errors. We analyzed the participant’s path (ie, steps) through the attempted completion of each task. Any deviation that occurred in the attempt to complete the task (eg, participant clicks on an incorrect menu item or participant struggled to get the user manual out of the box) was coded as a “use error.” For task 2, we specifically noted any instances where participants’ workflow deviated from the expected workflow (see steps in Textbox 2). We analyzed the time spent on each task and if the tasks were (1) completed, (2) completed with difficulty, or (3) not completed (task failure). We then calculated the task success rate. A task was only scored as a success if the user was able to complete the task requirements on their own without facilitator assistance. We calculated participants’ rating of difficulty for each task and the SUS scores. One researcher listened to the audio recording of each debrief interview and created a comprehensive list of feedback (eg, longer battery life) organized into two categories (1) what participants liked about the device and (2) areas for improvement. A team of human factors researchers reviewed all identified errors and participant difficulties completing tasks to develop design recommendations to improve the device, app, and packaging prior to market release. We mapped each design recommendation to specific human factors design principles, using the list of design heuristics described in Barton et al [22], which combined the commonly used heuristics for medical device design [23], interactive systems [24], and medical documents [25].

## Results

### Overview

Table 1 shows the participant demographics for each round of usability testing. Table 2 depicts the task success rates, number of use errors, task durations, and difficulty ratings for each task and round of usability testing.

While we identified minimal safety concerns with the device, we identified numerous usability issues, which led to several design modifications. We outline the key issues identified from the usability testing in the following sections.

**Table 1.** Participant demographics for each usability testing round.

	Round 1 (intended users, n=5)	Round 2 (intended users, n=15)	Round 3 (nonintended users, n=21)
**Hearing loss, n (%)**			
None or normal hearing	0 (0)	0 (0)	18 (86)
Mild	2 (40)	7 (47)	0 (0)
Mild to moderate	1 (20)	2 (13)	0 (0)
Moderate	2 (40)	6 (40)	0 (0)
Severe	0 (0)	0 (0)	3 (14)
Tinnitus (ringing in ears), n (%)	0 (0)	0 (0)	5 (24)
Average age (years), n (range)	65 (57‐73)	66 (56‐79)	65 (52‐77)
Women, n (%)	2 (40)	8 (53)	13 (62)
**Education, n (%)**			
High school or General Educational Development	0 (0)	5 (33)	5 (24)
Associate’s or trade school	1 (20)	2 (13)	4 (19)
4-year college	2 (40)	3 (20)	7 (33)
Graduate or professional school	2 (40)	5 (33)	5 (24)

**Table 2.** Usability testing performance.

Measure and round		Task 1	Task 2	Task 3	Task 4	Task 5
**Task success rate, n (%)**						
	Round 1	3 (60)	2 (40)	2 (40)	5 (100)	3 (60)
	Round 2	12 (80)	10 (67)	7 (47)	15 (100)	10 (67)
	Round 3	10 (48)	10 (48)	—^a^	—	—
**Average task duration (minutes), mean (SD)** ^b^						
	Round 1	0.72 (0.3)	9.33 (0.5)	1.37 (0.6)	0.50 (0.2)	1.08 (1.1)
	Round 2	0.83 (0.5)	11.24 (4.0)	2.21 (0.9)	0.67 (0.6)	0.95 (0.8)
	Round 3	—	—	—	—	—
**Average difficulty rating, mean (SD)** ^c^						
	Round 1	4.6 (0.5)	3.4 (1.2)	4.2 (0.8)	5 (0)	5 (0)
	Round 2	4.87 (0.4)	3.27 (1.4)	3.73 (1.2)	4.53 (0.8)	4.67 (0.6)
	Round 3	4.71 (0.6)	4.43 (0.9)	—	—	—
**Number of use errors per participant, n**						
	Round 1	0	4	0	0	0
	Round 2	0	4.9	0.5	0.6	0
	Round 3	0	0.7	—	—	—

a Not applicable.

b Only includes times for participants who successfully completed the task.

c Scale from 1 to 5 (1=very difficult, 5=very easy).

### Package Labeling

Overall, we found minimal safety issues with the set up and use of the device across all 3 rounds of testing. The one issue that presented potential, albeit minimal, concerns to safety was related to the accessibility of intended use and contraindications to use of the device. We found that the font size and color on the outside of the box made the intended use of the device hard to see and read (right side of Figure 1). Few participants saw or read this information on the box, and several participants indicated that they would not know the device was a hearing aid based on the outside of the box, stating *“*to know they are hearing aids, off the bat, I would have walked right by them.”

A total of 3 participants in usability testing rounds 1 and 2 failed task 1 (read outer label) since they could not determine if it was appropriate for them to use the product based on the outside of the box. We identified the same problem with nonintended users of the device, with over half of the participants in round 3 stating that they did not know that the device was a hearing aid from the labeling on the outside of the box. All 3 of the participants with severe hearing loss and 3/4 of participants with tinnitus said (incorrectly) that the device would be appropriate for them to use based on the labeling on the outside of the box.

### Device Pairing

**Figure 2.**
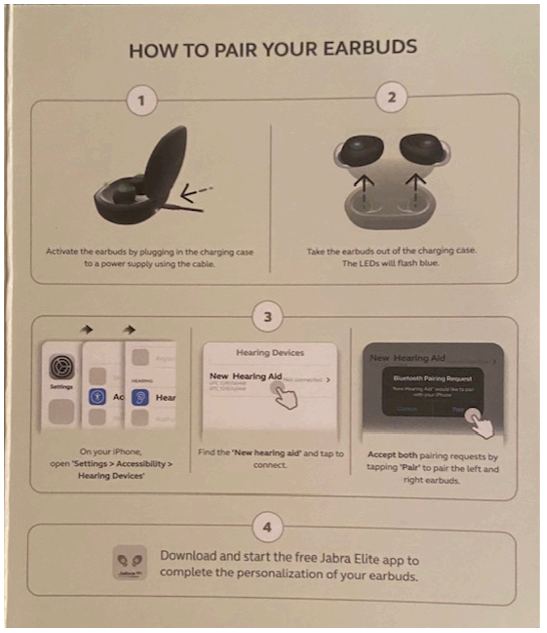
Quick guide instructions (December 2020 version—round 1 testing).

**Figure 3.**
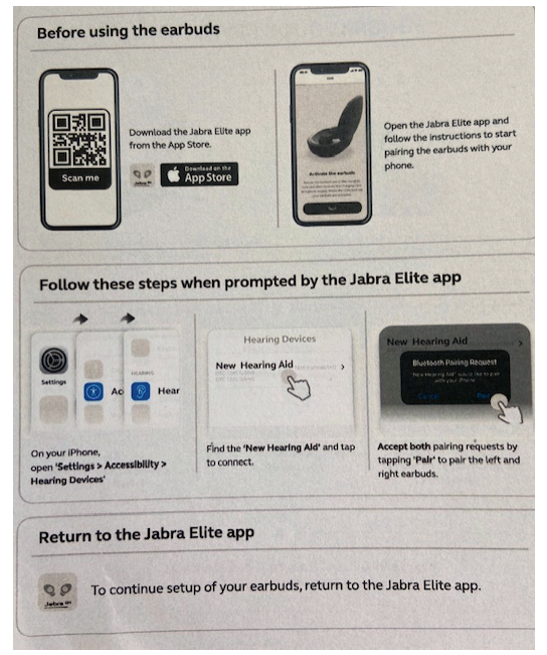
Quick guide (February 2021 version—round 2 testing).

The primary usability challenge with the device was setting up and pairing the hearing aids to the iPhone (task 2). In round 1, a total of 3 of 5 participants failed this task, and a fourth participant had difficulty completing it. A common challenge was that participants did not know that they needed to return to the app to complete the hearing aid set up after pairing the hearing aids to the iPhone. Rather, participants thought they were done setting up the device once they followed the Quick Guide pairing instructions (see Figure 2). The only participant to complete the task easily followed the workflow: open the Jabra Enhance app → pair the hearing aids using the iPhone → return to the app to complete the set up. As a result, we recommended design changes to the Quick Guide prior to round 2. As shown in Figure 3, the Quick Guide was revised to inform users to first go to the Jabra Enhance app before pairing the hearing aids to the phone. We further emphasized that users should return to the Jabra Enhance app after pairing to finish set up.

Following these design changes, task 2 success rate increased from 40% (2 out of 5 in round 1) to 67% (10 out of 15 in round 2). Yet, there were still several use errors during pairing and setting up the earbuds in round 2. For instance, 2 (13%) participants did not understand that they needed to go to an app to set up the earbuds. Several participants had trouble reading the Quick Guide instructions as the text was too small (see Figure 3). A total of 3 participants did not know what to do after pairing the earbuds to the phone. Finally, it was unclear to participants which section of the iPhone settings to use to pair the hearing aids. Some participants had difficulty finding the “Accessibility” section in the iPhone’s system settings, and some participants then went to the “Bluetooth” rather than the “Hearing Devices” section to pair the hearing aids.

### Distinguishing the Right and Left Earbud

Participants had difficulty determining which earbud was for the right versus the left ear. Several participants stated that they saw a “R” and “L” on the charging case, but not on the earbuds themselves. For instance, one participant stated, *“*so, there’s not a right and a left?” while another participant said, *“*I don’t see a left and right, they don’t indicate that right?” Some participants were eventually able to identify the “R” and “L” markings on the earbuds stating,

*Ah, there’s an R on the end, that’s really hard to see. I’d make that bigger...yeah that’s really hard to see.*

The inability to distinguish the right from the left earbud resulted in several use errors, including difficulty placing the earbuds in the ears and difficulty placing the earbuds back in the charging case.

### User Manual

**Figure 4.**
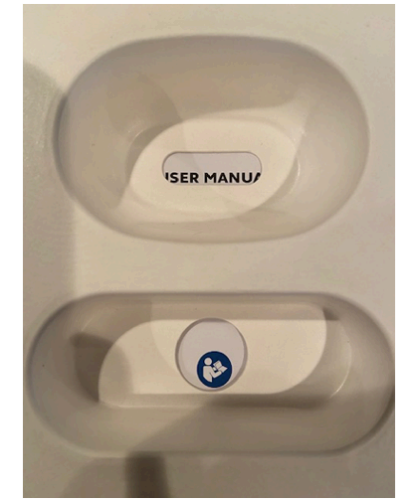
User manual cutout in box.

The user manual contained information about the intended use and about most of the hazards of device use. The location of the user manual at the bottom of the box led to several use errors. Several participants were not able to find the manual. Finding the user manual was challenging since the text “User manual” was partially covered by the cardboard insert (see Figure 4). One participant said,

*it’s kind of hidden though...that’s a little deceiving maybe, because I would have looked at this first.*

Another participant asked, *“*Where are the directions?*”* In total, 5 (33%) participants, all of whom identified the user manual, struggled to get it out of the box. Several more participants never took the manual out of the box; it is possible that these participants could not find the manual but did not verbalize this. Additionally, in round 1 testing, we found that there was not an entry in the table of contents to indicate on which page the “intended use” and “health and safety information” was within the user manual. This was corrected prior to round 2.

### Product Design

Overall, participants were very positive about the device. They liked the size and design of the charging case (eg, small and easy to put in a pocket or purse), and they liked that the device was rechargeable, and thus did not require batteries. Participants liked the sleek and modern looking design stating, *“*it feels high-end” and said that the hearing aids were easy to place in their ears. They appreciated the different sized ear gels and the magnets that assured that the earbuds clicked into the correct charging position in the case. Participants were excited that they could purchase the device “off-the-shelf” and stated it would be helpful to use when they were in crowded rooms. Despite observed difficulties with setting up and pairing the hearing aids to the iPhone (task 2), participants reported that the app was easy to use. Finally, participants liked that they could adjust the hearing aids’ settings on their phone.

Participants recommended several areas for improvement. Most participants desired a longer battery life (which was 6 h at the time of testing) so that they could wear the hearing aids all day without needing to recharge them. They also recommended that technical terms be better explained in the manual and on the box. One participant recommended that the hearing aids come in more discreet colors (ie, skin colored) instead of black. Participants recommended some modifications to the front of the box saying,

*it should have more on the front about like if it’s better for a noisier environment or something, because I’d be more likely to use it if it said, “will improve your hearing in a crowded environment” because I have a very hard time hearing when there is background noise.*

Some participants also found the contrast of white and red text on a gray background hard to read.

### Knowledge Assessment

Table 3 details the scores for each of the 10 knowledge assessment questions across all 3 rounds of testing. Participants who used the user manual performed better overall compared to those who did not access or refer to the user manual. The user manual appeared to help the most on question 9—whether someone who had a recent episode of dizziness should use the earbuds.

**Table 3.** Knowledge assessment (percent correct answers).

#	Question topic	Round 1 (n=5), n (%)^a^	Round 2 (n=15), n (%)^b^	Round 3 (n=21), n (%)^c^
1	Tinnitus	1 (20)	13 (87)	13 (62)
2	Sinus infection	0 (0)	15 (100)	15 (71)
3	Severe hearing loss	4 (80)	8 (53)	5 (24)
4	Rapid hearing loss	1 (20)	11 (73)	11 (52)
5	Preference for earbuds	1 (20)	12 (80)	15 (71)
6	Laryngitis	3 (60)	4 (27)	7 (33)
7	Sore in ear	5 (100)	13 (87)	17 (81)
8	Ear wax	3 (60)	12 (80)	11 (52)
9	Dizziness	4 (80)	9 (60)	11 (52)
10	Ear infection	5 (100)	15 (100)	21 (100)

a Average percent correct: 50%.

b Average percent correct: 75%.

c Average percent correct: 60%.

### Design Recommendations

Based on the identified usability issues, we recommended 9 design changes to the earbud system and its packaging (Table 4). We rated these recommendations as “high,”“medium,” or “low” priority. A total of 4 of the proposed changes (items 1, 2, 3, and 7 in Table 4) were implemented prior to subsequent testing rounds and all but 2 (items 4 and 5) were implemented prior to initial product release. Each design recommendation corresponds to a human factor’s design principle (descriptions of the design principles can be found in Barton et al [22]).

**Table 4.** Design recommendations derived from the results of 3 rounds of usability testing.

#	Priority	Addressed before round 2 testing?	Addressed before product release?	Design recommendation	Corresponding human factors principle
1	High	Yes	Yes	Increase the font size of the text in the Quick Guide	Readability (font and capitalization)
2	High	Yes	Yes	Revise the Quick Set-up Guide and user manual to clearly show the following steps: Download Jabra Enhance app from app store Open the Jabra Enhance app (show a picture of the app) Register and set up the hearing aids following the steps on the app Pair the hearing aids with the iPhone (show pictures to show the steps of going to settings > accessibility > hearing devices), select “New Hearing Aid” device, accept both pairing requests Return to the Jabra Enhance app (show picture of returning to app)	Organization (order)
3	High	Yes	Yes	Add a section in the user manual’s table of contents for “Intended use and warnings”	Organization (navigation tools)
4	High	No	No	Add a screen in the app that lists the contraindications of use (eg, severe hearing loss, tinnitus, and dizziness). This could be a screen after the registration in the app that participants acknowledge they understand reasons they should not use the device.	Content (emphasis)
5	Medium	No	No	Increase the font size of text about contraindications and intended use on the outside of the box.	Readability (font and capitalization)
6	Medium	No	Yes	More clearly indicate on the box that the device is to support those with hearing loss. It may be helpful to add the below text from the user manual, page 1, somewhere onto the outside of the box: “This product may help you, if you: Strain to follow conversations in both quiet and noisier environments Miss important information during conversations Have trouble hearing at a distance Have trouble understanding the television or telephone calls”	Content (clarity of content)
7	Low	Yes	Yes	Revise the app name and other aspects in the app and user materials to have a consistent term for the Jabra earbuds, for example, “Jabra Elite,” “Jabra earbuds,” and “Jabra hearing aid.”	Comprehensibility (terminology)
8	Low	No	Yes	Make the cut-out in the white box bigger so that the entire “User Manual” text can be seen (Figure 4), and add text directing users that the user manual is under the white box. Could say “User Manual” with an arrow pointing below the box.	Readability (layout and position)
9	Low	No	Yes	Add a note on the “manual pairing” page of the user manual that participants should refer to the “first time use” section if it is their first time setting up the device: “if this is the first-time use, please refer to page X.”	Organization (navigational tools)

### Device Packaging

We recommended that the warnings and intended use information on the outside of the box be better emphasized (eg, larger font size and more color contrast) to ensure appropriate use. Due to participants’ difficulty finding the user manual and getting it out of the bottom of the box, we recommended placing the user manual on the top so the user sees and touches it before accessing the product. This could improve safety as the user manual contains most of the warnings and intended use information about the device.

### Pairing the Device

To better support the pairing of the device to the smartphone, we recommended that the Quick Guide be revised. Despite modifications after round 1 testing, participants still could not read or follow the Quick Guide instructions due to the small text size. We recommended that the font size in the Quick Guide be significantly increased given the older population intended to use this device. We also recommended modifying the Quick Guide to clearly outline the pairing steps (eg, go to “Settings” then “Accessibility” then “Hearing devices”). As participants did not know to return to the app to complete set up after pairing the earbuds to the iPhone, we recommended that the steps in the Quick Guide be more clearly sequentially marked (eg, 1, 2, 3,... or a, b, c,...).

## Discussion

### Principal Findings

In this study, we evaluated the safety and usability of an over-the-counter hearing aid for people with mild to moderate hearing loss. We conducted usability testing with 20 intended and 21 nonintended users of the device. We identified numerous usability issues hindering the optimal use of the hearing aid, and generated design recommendations to mitigate use errors and improve the overall design of the device.

This work expands our understanding of the safety and usability of novel, over-the-counter hearing devices [18,26]. Overall, we found minimal safety-related issues with the device. The primary safety risk is that the diagnosis and treatment of a serious condition, such as sudden deafness due to infection, may be delayed due to the lack of medical screening prior to the device use [27]. Consistent with this, the main challenge we found was related to the lack of understanding by nonintended users, including those with tinnitus and severe hearing loss, that they should not use the device. This issue underscores the importance of ensuring that device contraindications and safety warnings are clearly displayed and legible, preferably on the outside packaging. Further, redundant messaging should be incorporated; we recommended a verification dialog in the phone app during device set up to ensure that users understood device use contraindications.

Usability testing identified numerous usability issues with the device, which largely related to violations of human factors design principles. Some examples of violations of the principle, readability (font and capitalization), were the small font size of the contraindications on the outside of the box, the Quick Guide, and the markings of “L” and “R” on the hearing aids. We also identified issues with comprehensibility (terminology), as the device was referred to by different terms (eg, “Elite” and “Rogue”) throughout the packaging and app. Based on our design recommendations, there were significant improvements in the usability of the device between the testing rounds. Human factors design principles [23,24], including those developed specifically for patient-facing medical documents [22,25], should be incorporated into the design of patient-facing medical devices. In hearing care, specific design guidelines for older adults (eg, use 12-14 pt font sizes, write in third-sixth grade level) [28] should be followed to maximize the ease of use of these devices given the substantial diversity in patients’ physical and cognitive attributes and abilities, experience, and background.

We observed appreciable tension between the goals of designing for safety and designing for ease of use, aesthetics, and marketability. For instance, from a safety perspective, the device’s contraindications for use (eg, tinnitus) should have been displayed in a large font such that they covered much of the back of the box. However, this would not be aesthetically appealing and there were concerns that it would deter customers from purchasing the device. Similarly, from a safety perspective, we recommended adding a hard stop in the app setup to ensure that the user understood the intended use of the device. However, this would have decreased the ease of pairing the earbuds to the phone. With over-the-counter medical devices, both product safety and aesthetic appeal are important [16] and it can be challenging to achieve both concurrently resulting in trade-off decisions. For devices with greater safety risks, safety should be prioritized.

We found that participants were excited about an over-the-counter hearing aid and appreciated this product’s design attributes. Similar to prior studies [18,26], participants liked the earbuds’ discreet size and form factor, which made hearing aid use less obvious, especially given the growing general use of earbuds for listening to music or talking on the phone. Participants also liked that the device did not require batteries and that it had a small carrying case that could easily fit in a pocket or purse. Our study identified excitement about the device from the intended user population, which may result in increased access to and use of hearing aids.

We uncovered challenges in conducting usability testing with nonintended users of the device. One goal of the FDA device approval process is to identify areas of foreseeable misuse including use of the device by unintended users [11]. This is a unique issue with over-the-counter devices, as there is no clinical mediation to ensure appropriate use. During testing, we found that only 50% of nonintended users were able to determine that they should not use the hearing device. While the design of the device and packaging likely contributed to this, another challenge is that participants may have assumed they should use the device since they were recruited to participate in the study. In essence, participants may have been “tricked” into thinking they should use the device due to the nature of usability testing. Future research should explore additional methods or approaches for eliciting information from nonintended users of over-the-counter medical devices.

Another limitation of this study is that the findings are based on users’ interaction with the device over only a short period of time; it is possible that as users learn to use the device, ease of use would increase or alternatively, new usability issues may emerge over prolonged use that we did not observe. We only used iPhones for testing and some of the participants were Android users. Despite diversity in participants’ age, hearing ability, comfort with technology, and education level, our sample may not have included the full range of end user cognitive abilities, experiences, and backgrounds. In the real environment of use, older adults may rely on adult children or other family members to assist with device pairing and set up; our study highlights the usability issues encountered by older adults setting up the device on their own, without assistance. We also did not test actual use of the hearing aid to enhance participant hearing so we cannot draw conclusions on the function of the hearing aid technology. Future studies should evaluate the use of the hearing aid in the real environment over time to determine if it supports users’ needs. There is increasing evidence, however, demonstrating efficacy of over-the-counter hearing aids in comparison to prescriptive devices [29,30].

### Conclusions

This study describes the safety and usability of an over-the-counter hearing aid for adults with mild to moderate hearing loss. Based on the usability testing, we proposed human factors design recommendations to enhance the usability and safety of the device. For instance, the intended user group and contraindications for use (eg, tinnitus) should be clearly displayed on the outside of the box, as well as throughout the set up materials. Clear step-by-step guidelines should be available to support user set up and pairing of the device. We identified challenges to the design and testing of direct-to-consumer devices. As the population ages and technologies continue to pervade every aspect of our lives, the direct use of medical devices by laypersons will continue to expand. This study lays the foundation for future studies on best practices for the user interface design of direct-to-consumer medical devices.

## Supplementary material

10.2196/65142 Multimedia Appendix 1 Knowledge assessment.

10.2196/65142 Multimedia Appendix 2 System Usability Scale.

10.2196/65142 Multimedia Appendix 3 Interview questions.

## Abbreviations

FDA: Food and Drug Administration
SUS: System Usability Scale
