# Supplementary material for: Evaluating the Safety and Usability of an Over-the-Counter Medical Device for Adults With Mild to Moderate Hearing Loss: Formative and Summative Usability Testing
Source: JMIR Hum Factors. 2025 Jan 20;12:e65142. doi: 10.2196/65142 (PMC11769691; doi:10.2196/65142)
Supplement: Multimedia Appendix 1 [file humanfactors-v12-e65142-s001.docx]

1. Your family member has told you that they have been recently experiencing a worsening of the ringing in their ears (tinnitus). Based on the materials in front of you, is it appropriate for your family member to use this device?
2. A different family member currently has a right-sided sinus infection with fullness and decreased hearing in the right ear. He thinks that the Earbuds might help him to hear better. Based on the materials in front of you, should he start using this device?
3. A friend of yours has tried hearing aids in the past, but hates having to change the batteries all the time. Without her hearing aids, she is almost deaf – she cannot hear a word anyone is saying unless the other person yells. She thinks that the Earbuds might be right for her. Based on the materials in front of you, should she use the Earbuds for her hearing problems?
4. Another friend thinks these Earbuds might be just the thing for them because in the last month their hearing has rapidly gotten worse especially on their left ear. Based on the materials in front of you, should this friend try this device?
5. Another family member thinks that the Earbuds may help him to hear conversations in noisy rooms better but is reluctant as he might not like having things in his ears. Based on the materials in front of you, does his concern that he might not like having things in his ears change if he should try this device?
6. A friend has been sick from a bad laryngitis for week now. She wanted to start using the Earbuds, that she just bought. Based on the materials in front of you, does her recent laryngitis change how or whether your friend should use this device?
7. A friend has bought the Earbuds, but now accidently hurt his left ear. A small unhealed sore close to the ear canal is visible. Based on the materials in front of you, does this unhealed sore change how or whether your friend should continue to use this device?
8. Your family member has heard of the Earbuds and is considering buying these. You know she often and regularly goes to the doctor to get ear wax removed. Based on the materials in front of you, does her need to have ear wax removed by a doctor on a regular basis change how or whether your family member should start using this device?
9. Another family member sometimes feels very dizzy. He has bought and uses the Earbuds. He tells you that he yesterday got very dizzy and had to sit down. Based on the materials in front of you, does this recent episode of dizziness change how or whether he should use this device?
10. A friend developed a middle ear infection while on a vacation. It hurts a little bit, and she describes that her ear canal feels wet or moist. She got the Earbuds as a gift. Based on the materials in front of you, does this middle ear infection change how or whether she should start to use this device?
