# Supplementary material for: Evaluating the Safety and Usability of an Over-the-Counter Medical Device for Adults With Mild to Moderate Hearing Loss: Formative and Summative Usability Testing
Source: JMIR Hum Factors. 2025 Jan 20;12:e65142. doi: 10.2196/65142 (PMC11769691; doi:10.2196/65142)
Supplement: Multimedia Appendix 2 [file humanfactors-v12-e65142-s002.docx]

Please answer the following questions about the Earbuds product including its labeling and the associated phone application you just experienced.

|  | Strongly Disagree |  |  |  | Strongly Agree |
| --- | --- | --- | --- | --- | --- |
| I think that I would like to use the Earbuds frequently. | 1 | 2 | 3 | 4 | 5 |
| I found the product unnecessarily complex. | 1 | 2 | 3 | 4 | 5 |
| I thought that the Earbuds were easy to use. | 1 | 2 | 3 | 4 | 5 |
| I think that I would need the support of a technical person to be able to use this system. | 1 | 2 | 3 | 4 | 5 |
| I found the various functions in the Earbuds were well integrated. | 1 | 2 | 3 | 4 | 5 |
| I thought there was too much inconsistency in the Earbuds. | 1 | 2 | 3 | 4 | 5 |
| I would imagine that most people would learn to use the earbuds very quickly. | 1 | 2 | 3 | 4 | 5 |
| I found the Earbuds very cumbersome to use. | 1 | 2 | 3 | 4 | 5 |
| I felt very confident using the Earbuds. | 1 | 2 | 3 | 4 | 5 |
| I needed to learn a lot of things before I could get going with the Earbuds. | 1 | 2 | 3 | 4 | 5 |
