# Supplementary material for: Evaluating the Safety and Usability of an Over-the-Counter Medical Device for Adults With Mild to Moderate Hearing Loss: Formative and Summative Usability Testing
Source: JMIR Hum Factors. 2025 Jan 20;12:e65142. doi: 10.2196/65142 (PMC11769691; doi:10.2196/65142)
Supplement: Multimedia Appendix 3 [file humanfactors-v12-e65142-s003.docx]

Now to conclude, I would like to ask you a few questions about your experience using the device today. As a reminder, I was not involved in the development of this product so please be open and honest in your feedback on how we can make this better.

- First, what did you like about it?
- What did not make sense to you?
- How would you improve this?
- Would you use this product? Why or why not?
- Would you recommend this product to anyone in your life? Why or why not?
- Do you think the information on the outside of the box worked to make this product safer? Why/why not?
  - Is there anything you would change to make the labeling on the outside of the box safer?
- Do you think the information on the inside of the box and/or the app screens worked to make this product safer? Why/why not?
  - Is there anything you would change to make the information on the inside of the box and/or the app screens safer?
- Do you have any other comments or questions?
